# Case Report: Successful Treatment of Refractory Interstitial Lung Disease With Cyclosporine A and Pirfenidone in a Child With SLE

**DOI:** 10.3389/fimmu.2021.708463

**Published:** 2021-10-04

**Authors:** Linxia Deng, Yaxian Chen, Xiufen Hu, Jianhua Zhou, Yu Zhang

**Affiliations:** Department of Pediatrics, Tongji Hospital, Tongji Medical College, Huazhong University of Science and Technology, Wuhan, China

**Keywords:** systemic lupus erythematosus, interstitial lung disease, child, pirfenidone, cyclosporine A (CsA)

## Abstract

Interstitial lung disease (ILD) as an initial manifestation of lupus is rare, especially in young children. Here, we report a case of a 3-year-old boy who presented with fever, shortness of breath, and facial erythema. Clinical examination suggested a diagnosis of active systemic lupus erythematosus (SLE) with butterfly rash, anemia, positive antinuclear antibody, positive anti-double-stranded DNA, and hypocomplementemia. On retrospective review of the patient’s records, multiple chest computed tomography (CT) images showed non-specific interstitial pneumonia + organizing pneumonia pattern, with no further autoimmune work-up during the visit to a respiratory department. In our opinion, persistent interstitial pneumonia may be a clue to connective tissue disease. The patient received steroid treatment for 1 year, and the radiological and immunological resolution was noted. However, he still suffered from cough and dyspnea. After a 1-year follow-up, he was hospitalized again for SLE relapse. While continuing corticosteroid therapy, the patient was given combination therapy consisting of cyclosporine A (CsA) and monthly-pulse cyclophosphamide for 6 months, and decreased proteinuria was noted. However, the patient’s respiratory symptoms and pulmonary radiologic findings did not improve significantly. With continued steroid therapy, the patient was started on a daily regimen of CsA and pirfenidone. Both drugs were sufficiently effective to allow gradual reduction of steroid dosage. After 2 years of treatment, marked improvements in symptoms, pulmonary function and chest CT images were observed. Our experience with this case emphasizes that prompt work-up for connective tissue disease (CTD) should be considered in young children with ILD, and pirfenidone might be a useful add-on therapy with immunosuppressive agents for refractory CTD-ILD in pediatric patients. Nevertheless, further clinical trials including larger numbers of patients need to assess the efficiency and safety of this combination therapy for refractory CTD-ILD.

## Introduction

Systemic lupus erythematosus (SLE) is a chronic autoimmune disease that affects multiple organ systems and is associated with a broad spectrum of clinical manifestations. Pulmonary involvement is common among SLE patients, with reported frequencies ranging from 24–70%, whereas pulmonary involvement at the onset of lupus has been reported in only 2–3% patients (1, 2). Interstitial lung disease (ILD) as an initial manifestation of lupus has been reported in only a few adult and adolescent cases, but none in a child as young as 3 years old (2–4).

ILDs are a diverse group of chronic lung disorders characterized by inflammation and/or fibrosis that result in thickening and distortion of the alveolar wall with consequent impairment of gas exchange. Management of connective tissue disease-associated interstitial lung disease (CTD-ILD) is challenging, largely due to the significant heterogeneity between CTD-ILD subtypes, as well as the lack of data from randomized controlled trials (RCTs) to guide management. Although effective, relying on conventional immunosuppressive agents including corticosteroids, cyclophosphamide, mycophenolate, etc. For the treatment of CTD-ILD alone is inadequate, and thus the search for novel agents continues. Recently, anti-fibrotic agents have been employed to treat CTD-ILD. However, to date, the efficacy of small-molecule anti-fibrotic agents has not been evaluated in pediatric patients. Here we report the efficacy and safety of combination therapy consisting of cyclosporine A (CsA) and pirfenidone in a 3-year-old SLE associated ILD patient.

## Case Presentation

A 3-year-old Chinese boy was referred to our hospital when he presented with fever, shortness of breath, and facial erythema. The patient was the first child of non-consanguineous parents and was delivered at 38 weeks after an uneventful pregnancy. His birth weight was 3400 g and his length was 51 cm. There were no signs or symptoms of congenital infection. His parents denied any family history of autoimmune disease. His past medical history revealed that he had been hospitalized often with pneumonia and needed oxygen support at night during sleep for 3 years since he was 3 months old. At that time, he did not have the typical symptoms and signs of lupus, such as rashes, hair loss, light sensitivity, joint pain, anemia, etc.

**Figure 1:**
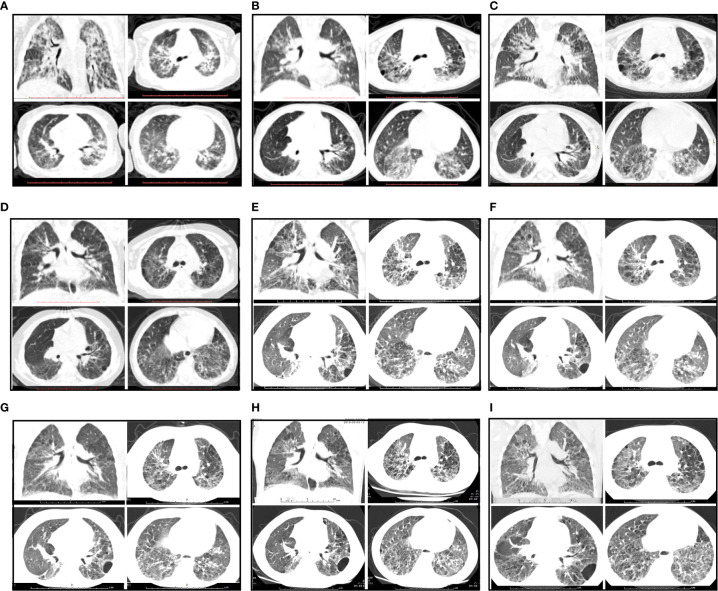
Radiological features during follow-up. **(A–C)** CT images showing a NSIP + OP pattern and reticular and ground-glass opacities along the bronchovascular bundles with patchy air space consolidation before prednisone treatment. **(A)** The first chest CT scan at the age of 3 months. **(B)** The subsequent CT scan at the age of 2 years. **(C)** The chest CT scan on admission (3 years of age). **(D)** CT images showing improvement after prednisone treatment for 3 months. **(E)** CT image showing progressive changes upon relapse of SLE after prednisone treatment for 1 year. **(F)** CT image showing no significant improvement after treated with prednisone plus CsA. **(G–I)** CT images showing improvement of NSIP after treatment with prednisone combined with CsA and pirfenidone for 7 months, 13 months and 26 months, respectively.

On admission, physical examination revealed a body temperature of 39°C, a heart rate of 116 beats/min, a respiratory rate of 35 breaths/min, a blood pressure of 92/58 mmHg, and oxygen saturation (SpO2) of 98% (room air). Three-concave sign, facial butterfly erythema, neck lymphadenopathy, and clubbed fingers were observed. The patient had no excess hair loss, oral ulcer, or arthritis. His parents reported his diminished exercise tolerance. On auscultation, loud crackles were audible over both lungs. The rest of the examination was unremarkable. Blood test showed a hemoglobin level of 103 g/L, platelet count of 206×10^9^/L, and white blood cell count of 10.38×10^9^/L with 60.5% neutrophils, 32.6% lymphocytes, and 5.7% monocytes. He had a C-reactive protein (CRP) level of 43.3 mg/L and an erythrocyte sediment rate of 107 mm/h. Urine analysis, serum biochemistry profile, serological antibody testing for common respiratory pathogens, and blood culture did not reveal any abnormalities. Immunologic testing was positive for antinuclear antibodies (ANA homogenous type 1:1000), including those to double-stranded (ds)DNA, with low complement levels of C3 (0.29 g/L, normal range 0.79–1.52 g/L) and C4 (0.03 g/L, normal range 0.16–0.38 g/L), suggesting a diagnosis of SLE. Lung function tests could not be completed due to his poor cooperation. Echocardiography demonstrated that the left ventricle was of normal size with normal systolic function, and no left ventricular diastolic dysfunction was detected. The estimated pulmonary artery pressure was 41 mmHg, suggesting pulmonary artery hypertension, and the ejection fraction was 68%. High-resolution computed tomography (HRCT) scanning showed reticular and ground-glass opacities along the bronchovascular bundles with patchy air space consolidation following the nonspecific interstitial pneumonia (NSIP) with organizing pneumonia (OP) pattern (**Figure 1C**). On review of the patient’s previous medical records, it was found that the patient had been prone to pneumonia since the age of 3 months, and the initial and subsequent chest CT images all showed reticular and ground-glass opacities (**Figures 1A, B**).

To investigate the possibility of congenital immunodeficiency, whole-exome sequencing and copy number variation detection of the proband was performed. Two paternally inherited heterozygous mutations (c.3172T>C, p.Y1058H and c.3178G>C, p.V1060L) in *CFH* exon 20 and a *de novo* heterozygous deletion of the entire *CD19* gene were detected. However, the levels of serum complement factor H, serum immunoglobulins, and CD19 molecule in B lymphocytes were normal. Therefore, we considered that the patient did not have congenital immunodeficiency or STING-associated vasculopathy.

**Figure 2:**
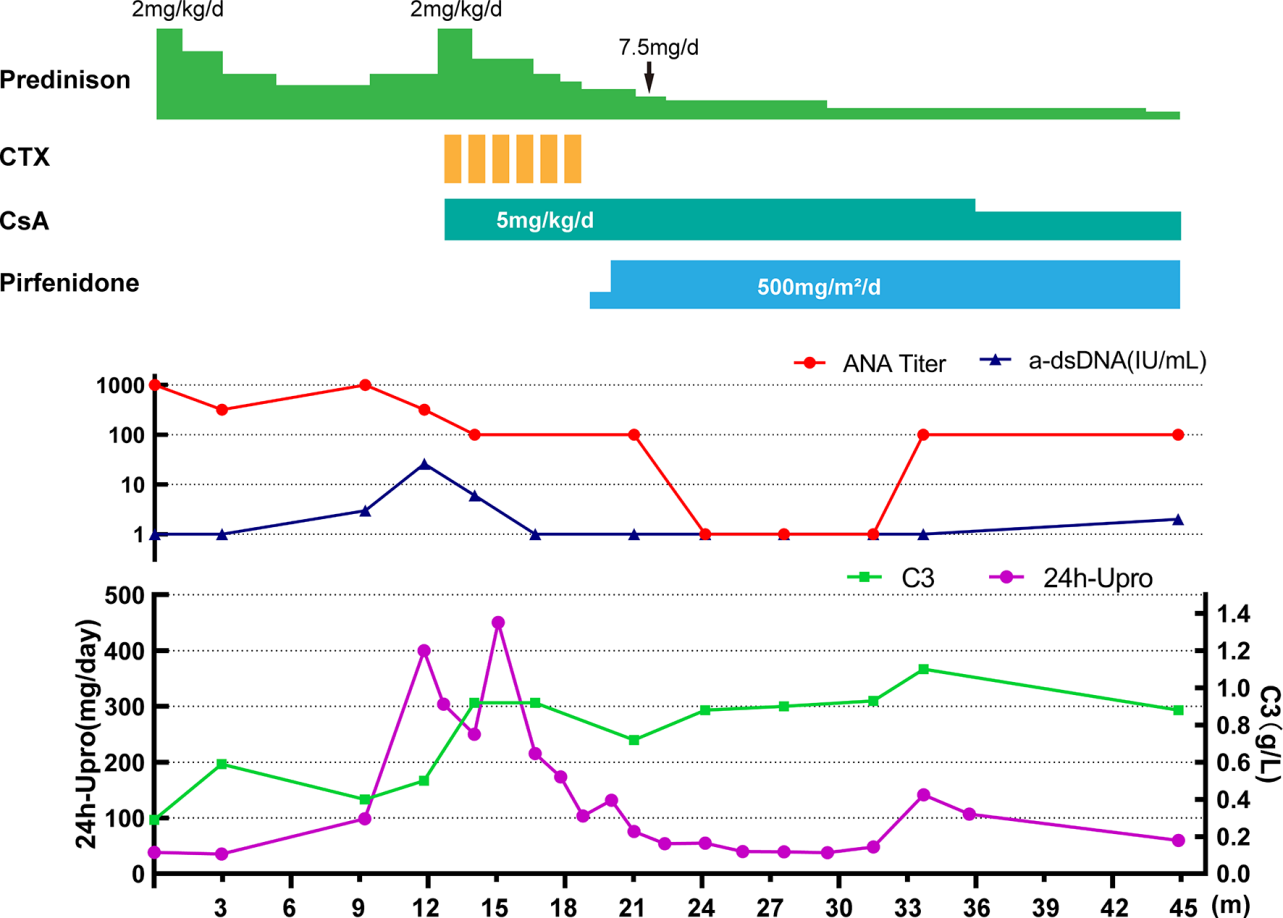
Clinical course of the patient. CTX, cyclophosphamide; CsA, cyclosporin A; 24h-Upro, 24-hour urinary protein quantity.

Based on the above findings, a diagnosis of SLE (SLEDAI score 7) with ILD was established, and the patient was started on a prednisone 2 mg/kg/day (**Figure 2**). With radiological and immunological resolution indicating remission (**Figures 1D** and **2**), the steroid dosage was tapered gradually during outpatient follow-up. Nonetheless, the boy was still hospitalized two more times, with similar respiratory symptoms (nonproductive cough and shortness of breath).

**Figure 3:**
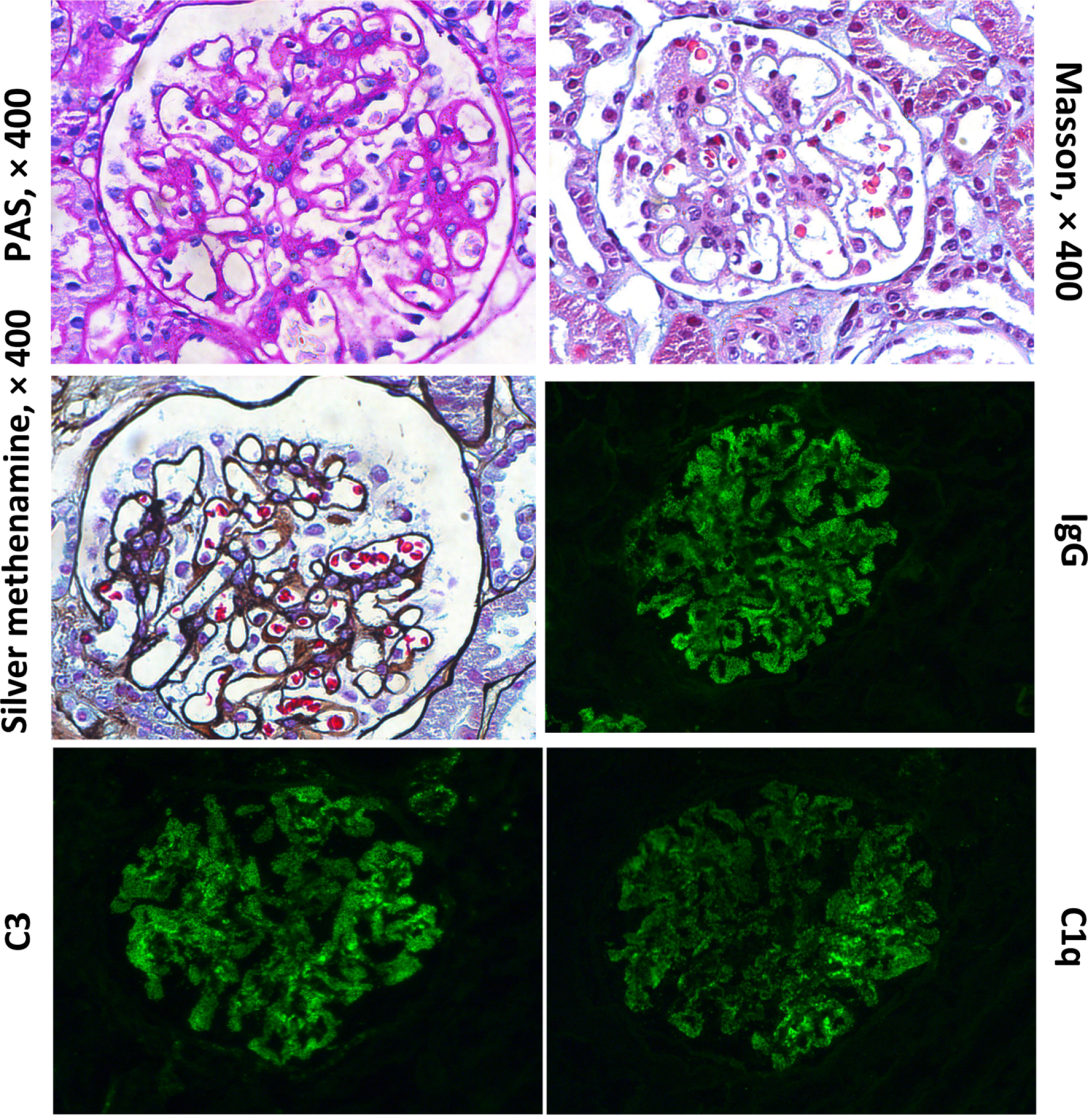
Histological findings from renal biopsy specimen. Renal biopsy showed one segmental glomerulosclerosis on 50 glomeruli with “full house” pattern depositions within capillary loops.

After 1-year of follow-up, the patient was hospitalized again for recurrent facial and finger erythema with ulcer, nonproductive cough and eyelid edema. On this admission, the patient had a hemoglobin level of 152 g/L, platelet count of 235×10^9^/L, and white blood cell count of 5.19×10^9^/L with 74.4% neutrophils, 16.4% lymphocytes, and 9.2% monocytes. He had moderate proteinuria (400 mg/24 h). A renal biopsy was performed, and histological examination revealed typical lupus nephritis (class V) (**Figure 3**). CT scanning of the brain showed multiple contrast enhancing lesions of variable sizes in the bilateral basal ganglia and bilateral frontal parietal lobe. Chest HRCT showed progressive changes (**Figure 1E**). Immunologic testing was again positive for ANA (homogenous type 1:320) and anti-dsDNA (26 IU/ml, normal range ≤ 2 IU/ml), with low complement levels of C3 (0.5 mg/L, normal range 0.79–1.52 g/L) and C4 (0.08 mg/L, normal range 0.16–0.38 g/L), suggesting relapse of SLE (SLEDAI score 16). The patient was continued on steroids, with a plan to start CsA (5 mg/kg/day, lowest concentration 100–150 µg/L) with monthly-pulse cyclophosphamide (CTX, accumulated dose 150 mg/kg). By 6 months later, his proteinuria and nonproductive cough had disappeared, but he still suffered from dyspnea. Repeat lung scans showed no significant improvement (**Figure 1F**). Pulmonary function tests were successfully performed and reveled a reduced forced vital capacity (FVC) of 61%, a forced expiratory flow at 50% of vital capacity (FEF 50) of 0.40, a FEF 75 of 0.14, and a maximal mid-expiratory flow (MMEF) of 0.26, which reflected a moderate restrictive ventilatory pattern with small airway dysfunction (**Table 1**). Steroids and CsA were continued, and pirfenidone treatment also added at an initial dose of 125 mg/m^2^/day and increased to 500 mg/m^2^/day within 1 month. During the next 2 years of follow-up, the patient’s respiratory symptoms (e.g., nonproductive cough and shortness of breath) disappeared, and his exercise tolerance returned to normal. Furthermore, radiologic findings and pulmonary function improved (**Figures 1G–I** and **Table 1**).

**Table 1:** Results of pulmonary function tests.

Data*	Before pirfenidone treatment	After 7 months of pirfenidone treatment	After 13 months of pirfenidone treatment	After 26 months of pirfenidone treatment
FVC (% predicted)	61	62.9	77.9	75.4
*FEV_I_ (% predicted)	0.47 (53.4)	0.60 (68.8)	0.7 (80.0)	0.99 (82.8)
FEV_I_/FVC (%)	74.72	93.18	87.59	93.03
FEF50 (% predicted)	26.3	86	125.6	105.6
FEF75 (% predicted)	17.4	36.9	49.4	58.6
MMEF 75/25	20.8	60.9	92.5	81.2

*Numbers within parentheses are percentages of predicted value. FVC, forced vital capacity; FEV1, forced expiratory volume in 1 second; FEF, forced expiratory flow; MMEF, maximal mid-expiratory flow.

## Discussion

Pulmonary involvement in the form of ILD occurs much less frequently in SLE than in other CTDs, with reported incidence rates of 3–13% in cases of SLE (5, 6). The onset is usually insidious, although it may be preceded by an episode of acute pneumonitis. Chronic nonproductive cough, dyspnea, recurrent pleuritic chest pain, and decreased exercise tolerance are the usual complaints. Moreover, early-onset SLE is a rare disease with variable clinical manifestations (7). Consequently, SLE with ILD as an initial manifestation in a very young child could be easily missed by pediatric respirologists. Our experience with the present case emphasizes that in very young children presenting with recurrent respiratory symptoms, such as nonproductive cough or dyspnea, early-onset SLE with ILD should be considered and ruled out.

CTD-ILD is initially diagnosed mainly based on chest radiographs. HRCT is extremely helpful in the diagnosis of ILD and classification according to the International Classification Statement/Guideline for idiopathic interstitial pneumonias (IIP). In patients with IIP, IP patterns are important for predicting prognosis and selecting therapies (8). A previous study demonstrated that the most frequent SLE-ILD pattern on both HRCT and surgical lung biopsy/autopsy specimens is “unclassifiable”, including the NSIP+OP pattern and others in adults, and this category of “unclassifiable” is considered to represent the heterogeneity of lung inflammation and fibrosis (6). Enomoto et al. concluded that patients with a NSIP+OP pattern on HRCT have a better prognosis than those with the NSIP only pattern, because the predominant milieu in the lung of these patients is inflammatory (6). Based on pulmonary CT images consistent with the NSIP+OP pattern in the present case, we attempted to suppress autoimmune inflammation *via* immunosuppressive therapy first.

To treat inflammation in CTD-ILD patients, the use of steroids plus immunosuppressants is a common regimen. From recent clinical trials on CTD-ILD, CTX is one of the most evidence-supported immunosuppressants. It was shown to improve pulmonary function by 49.2% to 61.5% during the first 1-year follow-up and also to improve dyspnea and pulmonary CT appearance (9, 10). Mycophenolate mofetil (MMF) is also approved for the treatment of ILD. Based on our current understanding, the efficacy of MMF with respect to the improvement of FVC, DLco (diffusing capacity of the lungs for carbon monoxide) and several patient-reported outcomes is comparable to that of cyclophosphamide (11, 12). MMF does not perform better than CTX in patients with systemic sclerosis-related ILD. The efficacy of CsA in ILD also has been reported, with studies concluding that CsA should be used early to obtain a favorable response (13, 14). Furthermore, in a combination therapy regimen, CTX, CsA, and prednisolone were shown to act synergistically to improve the survival rate of patients with dermatomyositis-associated acute/subacute interstitial pneumonia (15). Unfortunately, the patient in the present case did not respond well to this combination therapy regimen during a 6-month follow-up. Although his proteinuria and nonproductive cough disappeared, he continued to suffer from dyspnea. To improve treatment efficacy, a combination of immunosuppressant and anti-fibrotic agent could be a reasonable approach.

To stop the fibrotic process (including pulmonary fibrosis, hepatic cirrhosis, renal fibrosis, etc.), small-molecule anti-fibrotic agents such as pirfenidone, and nintedanib have been approved in the last decade. Pirfenidone, 5-methyl-1-phenyl-2-(1H)-pyridone, is a broad-spectrum oral antifibrotic drug that modulates the action of cytokines such as transforming growth factor (TGF)-β, tumor necrosis factor (TNF)-α, platelet-derived growth factor (PDGF), interferon (IFN)-γ, fibroblast growth factor, interleukin (IL)-6, IL-18, etc. (16, 17). It was approved for the treatment of idiopathic pulmonary fibrosis (IPF) by the US Food and Drug Administration in 2014 (18). Treatment with pirfenidone has been shown to lower the risk of respiratory-related hospitalization, slow the worsening of patient-reported breathlessness, and reduce the rate of lung function decline in IPF patients (18–20). Currently, various clinical trials are planned or ongoing to evaluate the safety and efficiency of pirfenidone in patients with CTD-ILD (21). Data from a clinical trial of pirfenidone for the treatment of pediatric neurofibromatosis type 1 and plexiform neurofibromas showed that pirfenidone is well-tolerated without notable toxicity, causing only minor adverse events that do not represent major health risks (22). However, at present, there is lack of data supporting the use of pirfenidone in pediatric CTD-ILD. The present case provided the first demonstration of the efficacy of combination therapy with pirfenidone and CsA for refractory CTD-ILD in a young child. Importantly, no adverse events were noted during follow-up. Our data indicate that CsA and pirfenidone likely work in synergy, and pirfenidone might be a useful add-on therapy with immunosuppressive agents for refractory CTD-ILD in pediatric patients. Nevertheless, further clinical trials including larger numbers of patients are needed to assess the efficiency and safety of pirfenidone for pediatric CTD-ILD.

## Data Availability Statement

The original contributions presented in the study are included in the article/supplementary material. Further inquiries can be directed to the corresponding author.

## Ethics Statement

The studies involving human participants were reviewed and approved by the Ethical Committee of Tongji Hospital. Written informed consent to participate in this study was provided by the participants’ legal guardian/next of kin.

## Author Contributions

LD, YZ, and JZ designed the study. LD, YC, XH, JZ, and YZ collected data and performed analyses. LD drafted the manuscript. YZ edited and revised manuscript. All authors contributed to the article and approved the submitted version.

## Funding

Science and Technology Planning Project of Wuhan (NO. 2016060101010041).

## Conflict of Interest

The authors declare that the research was conducted in the absence of any commercial or financial relationships that could be construed as a potential conflict of interest.

## Publisher’s Note

All claims expressed in this article are solely those of the authors and do not necessarily represent those of their affiliated organizations, or those of the publisher, the editors and the reviewers. Any product that may be evaluated in this article, or claim that may be made by its manufacturer, is not guaranteed or endorsed by the publisher.
